# Motivations for Caffeine Consumption in New Zealand Tertiary Students

**DOI:** 10.3390/nu13124236

**Published:** 2021-11-25

**Authors:** Saskia Stachyshyn, Carol Wham, Ajmol Ali, Tayla Knightbridge-Eager, Kay Rutherfurd-Markwick

**Affiliations:** 1 School of Sport, Exercise and Nutrition, College of Health, Albany Campus, Massey University, Auckland 0632, New Zealand; saskiastash@hotmail.com (S.S.); C.A.Wham@massey.ac.nz (C.W.); a.ali@massey.ac.nz (A.A.); T.Eager@massey.ac.nz (T.K.-E.); 2 Centre for Metabolic Health Research, Massey University, Auckland 0632, New Zealand; 3 School of Health Sciences, College of Health, Albany Campus, Massey University, Auckland 0632, New Zealand

**Keywords:** caffeinated product, caffeine supplement, caffeine literacy, chocolate, coffee: energy drink, tea

## Abstract

Caffeine-related health incidents in New Zealand have escalated over the last two decades. In order to reduce the risk of substance-related harm, it is important to understand the consumers’ motivations for its use. This is especially true for tertiary students who are presumed to be at a higher risk due to seeking out caffeine’s well-known cognitive benefits as well as the targeted marketing of such products to young adults. This study examined the habits and motivations for caffeine consumption in tertiary students in New Zealand. A previously validated caffeine consumption-habits (CaffCo) questionnaire was administered online to 317 tertiary students (*n* = 169 females), aged ≥16 years. Of the 99.1% of participants who regularly consumed caffeine, coffee (76.3%) tea (71.6%) and chocolate (81.7%) consumption were the most prevalent. Motivations for caffeinated-product consumption differed according to caffeine source. Tea was consumed for the warmth and taste, coffee was consumed to stay awake and for warmth, and chocolate, for the taste and as a treat. Marketing was not identified by participants as influencing their consumption of caffeinated products. Knowledge of motivations for caffeine consumption may assist in identifying strategies to reduce caffeine intake in those New Zealand tertiary students who regularly consume amounts of caffeine that exceed safe level.

## 1. Introduction

Many factors influence the consumption of foods and beverages [1] including those containing caffeine, such as expected outcomes (i.e., the functional qualities), as well as hedonic, environmental and sociocultural influences. Furthermore, reasons for intake may differ according to the caffeine source and the consumer population group. The theory of ‘planned behaviour’ [2] provides an explanation for why consumers’ motivations and expected outcomes are likely to predict and explain specific caffeine consumption behaviours. The proclaimed benefits of caffeine include increased alertness, endurance, and attention, as well as combating fatigue [3,4]. These desirable effects are likely to reinforce caffeine consumption, whereas negative effects such as anxiety, nervousness, and sleep disturbances are likely to discourage additional or continual caffeine consumption [4,5,6,7,8,9]. In addition, since genetics may affect whether an individual responds to caffeine in a positive or negative way, there will also be a genetic influence on the amount of caffeine consumed by an individual [10].

Caffeinated products are predominantly consumed according to their hedonic properties such as taste and temperature [4]. However, other factors also influence consumption; for example, the act of ‘going out’ for tea or coffee is a popular social activity, and in an environment where others are consuming caffeinated products, an individual may be more likely to consume caffeine in order to feel accepted [11]. Likewise, when ordering a caffeinated product, it may be perceived by some cultures as rude to decline and thus induces individuals to consume something they normally would not. 

Marketing of certain caffeinated products such as energy drinks, soft drinks and ready-to-drink alcoholic beverages (RTD) often targets specific populations such as young adults and adolescents [12,13]. However, whether this marketing actually influences the consumption of these products has yet to be determined. 

Nutritional knowledge may also affect caffeine intake. An increased awareness of the negative effects of consuming energy drinks has been associated with a decreased consumption [14]. However, this knowledge effect appears to be dependent on the type of consumer. Evidence has indicated that even if an advisory health statement was presented and read, existing beliefs, experiences and information from peers may have altered how consumers interpreted the message [15,16,17]. It has also been reported that, in general, the importance placed on the health message can differ according to gender, with males often making poorer food and beverage choices, as compared to females [18].

The desire for certain effects from caffeine, and therefore themotivations for consumption, can also differ between users. For example, athletes may use caffeine as an ergogenic aid to increase their performance during training or competition [19], whereas it has been reported that students are likely to consume caffeine to increase concentration [20], to stay awake or to combat fatigue [12,21,22]. 

Our earlier publication [23], from the same study described here, indicated that one-third (34.4%) of the 317 ≥16-year-old NZ tertiary (university) students surveyed consumed caffeine levels that were over the adverse effect level (3 mg·kgbw^−1^·day^−1^), with 14.3% being over the safe daily limit for caffeine (400 mg·day^−1^). Consumption of coffee and alcoholic RTDs were both associated with a greater risk of consuming an amount that exceeded the adverse effect and safe daily levels for caffeine [23]. Of particular concern was that most students (84.7%) reported experiencing adverse effects following caffeine consumption, with one-quarter indicating that the effects led to distress or negatively impacted on their life. However, those negative experiences did not result in the symptomatic participants reducing or discontinuing their consumption of caffeine [23]. Hence the motivations behind consumption appear to be overriding physical wellbeing in many cases, which goes against the expectation that negative effects would discourage consumption. It is important to establish the motivations for caffeine consumption by NZ tertiary students to better understand the factors leading to consumption that exceeds the safe limit in order to develop and implement caffeine-related risk-reduction strategies for this specific population group.

Therefore, the aim of this study was to investigate consumption habits associated with caffeine-containing products including coffee, tea, chocolate, and caffeinated-alcoholic beverages and establish the key motivations (e.g., functional, hedonic, sociocultural) for consumption of these products by NZ tertiary students. This knowledge, particularly that related to the products associated with the greatest risk of consumption exceeding the safe daily caffeine limit, may provide a the basis for developing a strategy to reduce caffeine intake in NZ tertiary students.

## 2. Materials and Methods

### 2.1. Study Design

This work was part of a larger nationwide study investigating New Zealanders and caffeine consumption, which included the amount of caffeine consumed, the related habits, the motivations for consumption, and the experiences following ingestion [23,24,25]. This work also assessed how specific caffeine-related genes influence consumption habits. The current work was part of a cross-sectional study: “Caffeine consumption habits, motivations and experiences of New Zealand tertiary students” [24] which collected data using a validated caffeine consumption-habits (CaffCo) questionnaire [25]. The study investigated caffeine-consumption habits (e.g., sources, amounts, frequency) from a convenience sample of tertiary students in New Zealand [23]. The reasons for consumption and non-consumption of caffeinated products are explored here.

### 2.2. Participants

A total of 317 tertiary students (*n* = 169 females), aged ≥16 years-old, voluntarily participated in this study. Consumption of caffeinated products was not a requirement for inclusion. 

Ethical approval was gained from the Massey University Human Ethics Committee: Southern A (Application 15/76). Participants provided written informed consent via a tick box incorporated into the beginning of the online questionnaire. As participants were recruited in person, they were not able to remain anonymous to the researchers. The data however, were completely anonymized, via the use of a unique identifier (six-digit numerical figure) assigned to each participant. 

### 2.3. Experimental Procedures

Between June and August 2016, an online survey was administered to participants, who were recruited via media releases in newspapers, posters, word of mouth, and in person at the time/place of data collection. Further information regarding data collection (i.e., dates, locations, and times) was provided to persons who expressed interest in the study. 

Participants were able to complete the questionnaire at the points of data collection (Massey University Albany, Massey University Palmerston North, and University of Auckland City campus) or at a separate location of their choice. If participants chose the latter, they were provided with their unique identifier code and a link to the questionnaire. 

Qualtrics (Version 3.673s, Qualtrics, Seattle, WA, USA, http://qualtrics.com, accessed 18 November 2017 ) online survey software [26] was used to administer the questionnaire. Screening questions were incorporated into the questionnaire to determine whether individuals met the inclusion criteria of being aged ≥16 years of age and being currently enrolled in either part-time or full-time study at a higher-education facility. To allow the results from this research to be aligned with the data from the 2008/2009 NZ Adult Nutrition Survey [27] persons aged under 15 years of age were unable to participate. 

### 2.4. Caffeine Consumption Habits (CaffCo) Questionnaire

The caffeine-consumption habits questionnaire (CaffCo) was a validated online questionnaire, designed to examine caffeine habits, consumption patterns, positive and negative experiences, and influences, across a variety of caffeinated products [25]. In this sub-study, data from the CaffCo was used to investigate consumption habits and explore the motivations behind caffeine consumption in tertiary students in NZ. 

Relevant to this part of the study, the CaffCo questionnaire was used to obtain information pertaining to what caffeine-containing products participants consumed and the reasons why respondents chose to consume or not consume a range of these products (e.g., tea, coffee, chocolate, cola drinks, energy drinks, caffeinated RTDs, sports supplements, caffeine tablets). The CaffCo questionnaire included decaffeinated coffee and caffeine-free cola drinks as separate product options allowing differentiation between consumption of caffeinated and de-caffeinated versions of a product. The main reasons given (according to accumulative percentage of agreement) for consuming or not consuming these products were explored using a four-point Likert scale (strongly agree, agree, disagree, strongly disagree). The most common reasons for consumption were divided into the categories of functional, hedonic, or sociocultural, as listed here, and other possible choices are listed as a footnote to Table 4. There were functional (i.e., “for energy”, “for mental energy”, “for physical energy”, “to improve physical performance”, “to stay awake”, “to wake up”, “for the alcohol content”, “because I know how much alcohol is in them”, “because they are cheaper than other alcoholic drinks”), hedonic (i.e., “for the taste”, “for the warmth”, “because they are cold and refreshing”, “to comfort and relax myself”, “as a treat drink”, “as a treat or luxury food”), and sociocultural (i.e., “with family”, “with friends”, “because others are drinking them”, “whenever one is offered to me”, “with takeaway food”,” because it is easily available”, “while travelling”).

### 2.5. Statistical Analysis

Data from the CaffCo questionnaire were exported from Qualtrics into Microsoft Excel (2013), screened for any missing information, and then imported into a statistics package (IBM SPSS Statistics for Windows, Version 25.0 Armonk, NY, USA) for statistical analysis. Participants were categorised into gender and age groups (16–18 years, 19–30 years, 31–50 years and ≥51 years). 

Categorical data were reported as frequency and percentage, and contingency tables were used to compare percentage consumption of the caffeine sources according to different demographic and participant characteristic groups. Statistical significance was indicated by a *p* value of < 0.05 for all tests.

For 2 × 2 contingency tables, the Fisher’s exact test was used if any of the expected counts were less than 5, the Yate’s continuity correction was applied if any of the expected counts were less than 10 but greater than or equal to 5, and the Pearson’s chi-squared test for independence was used if all expected counts were 10 or greater [28]. 

For contingency tables larger than 2 × 2, the Pearson’s chi-square test of independence was used under the condition that “no more than 20% of the expected counts are less than 5 and all individual expected counts are 1 or greater” [29]. The Fisher’s exact test was used if these conditions were not met. If these larger contingency tables (>2 × 2) reached significance, post hoc testing was carried out using multiple 2 × 2 contingency tables and the stepwise Holm-Bonferroni method; the odds ratio was also calculated to show the practicality of the significance.

## 3. Results

### 3.1. Participants

The final dataset consisted of 317 tertiary students, aged ≥16 years old. Socio-demographic data are shown in Table 1 [23]. Further participant characteristics were published elsewhere [23]. The majority of participants were female (*n* = 169, 53.3%), NZ European (*n* = 150, 47.5%), living with family (*n* = 174, 54.9%), not in paid employment (*n* = 211, 66.6%), non-smoking (*n* = 268, 84.5%), played sport (*n* = 189, 59.6%), and were aged 19–30 years old (*n* = 236, 74.4%). 

### 3.2. Consumption of Caffeine Sources according to Participant Demographics and Characteristics

Nearly all the participants (*n* = 314, 99.1%) reported regular consumption of caffeine-containing foods and beverages. Females were 2.42 times more likely than males to consume tea (79.9% vs 62.2%, *p* < 0.001), 1.75 times more likely to consume coffee (81.1% vs. 76.3%, *p* = 0.034), and 2.35 times more likely to consume chocolate (87.6% vs. 75.0%, *p* = 0.004; Table 2). There was no difference in the consumption of any other caffeine-containing products between males and females (all *p >* 0.05). 

The age ranges used here were based on those from the 2008/2009 New Zealand adult nutrition survey [27]. Although no association was seen between age groups and consumption of any of the caffeine sources (all *p* > 0.05; Table 2), relative to the 16–18-year-group, the 31–50 (*p <* 0.001) and 19–30 (*p <* 0.01) year age groups both had a higher estimated daily caffeine intake from coffee. Of the total participants, 38.5% (*n* = 122) reported co-ingesting caffeine and alcohol. Of this group, 71.3% co-ingested cola drinks and alcohol, 48.4% co-ingested energy drinks and alcohol, and 47.5% consumed caffeinated RTDs. 

Working and smoking status were associated with co-ingestion of caffeine and alcohol and energy drinks as well as alcohol (Table 3). Compared to those who did not smoke, participants who smoked were 3.43 times more likely to co-ingest caffeine and alcohol (63.8% vs. 34%, *p <* 0.001) and 3.88 times more likely to co-ingest energy drinks and alcohol (40.4% vs. 14.9%, *p* < 0.001). Compared to those who did not have paid employment, those with paid employment were 1.72 times more likely to co-ingest caffeine and alcohol (47.2% vs. 34.1%, *p* = 0.024) and 2.94 times more likely to co-ingest energy drinks with alcohol (30.2% vs. 12.8% *p* < 0.001).

There was also a trend (*p* = 0.059) found in the two younger age groups (i.e., 16–18, and 19–30 years) as being more likely to co-ingest caffeine and alcohol than the older age groups. No other participant demographic or characteristic was associated with the co-ingestion of either caffeine or energy drinks in addition to alcohol (*p* > 0.05).

### 3.3. Motivations for Consumption of Caffeinated Products

Of participants who consumed each caffeine source (Table 2), the principal factors influencing consumption are shown in Table 4. The main reasons that students reported for consuming tea were related to hedonic factors: “for the warmth” (92.6%), “for the taste” (89.5%), and comfort and relaxation (86.9%). 

Coffee consumers reported their primary reasons for consumption were due to functional effects such as increasing wakefulness: “to stay awake” (86.8%), “to wake up” (85.9%), and for energy (84.3%; mental energy 85.5%).

Consumers of chocolate reported their primary reasons for consumption were related to hedonic factors including “for the taste” (95.4%), as a treat food (88.8%), and for comfort and relaxation (79.6%).

Tertiary students also reported consuming cola drinks for their hedonic effects: “because they are cold and refreshing” (90.6%), “for the taste” (89.3%) and with takeaway foods (85.5%). 

Energy drink consumers reported doing so mainly for the functional effects of providing energy (90.6%; mental energy 84.3%) and increasing wakefulness: “to stay awake” (89.1%) and “to wake up” (85.2%).

Sociocultural reasons appeared to be the key motivation for tertiary students to consume caffeinated RTDs, with most participants who consumed them doing so “with friends” (91.8%) and “because others are drinking them” (78.7%). Functional factors around the alcohol content of these beverages were also common reasons for consumption: “for the alcohol content” (85.2%), “because I know how much alcohol is in them” (72.1%), and “because they are cheaper than other alcoholic drinks” (62.3%).

Of the participants who consumed caffeine-containing sports supplements (*n* = 21), most did so for the functional effects: “for energy” (86.3%), “for physical energy” (81.8%) and “to improve physical performance” (86.3%). They also reported consuming these supplements “as they are convenient to take” (59.1%).

The desire for functional effects was the primary reason motivating tertiary students to consume caffeine tablets (*n* = 10 participants). They reported doing so for energy (90.9%; “for mental energy” 90.9%, “for physical energy”, 54.6%) and increased wakefulness: “to stay awake” (81.8%), and “to wake up” (81.8%). “Convenience” (“as they are convenient to take”) was also a motivator reported by nearly two-thirds of caffeine-tablet consumers (63.7%).

Advertising (“because I feel that I am influenced by advertising”) did not appear to be a key factor influencing participants consumption of caffeinated products (0–29.3%; caffeine tablets and chocolate respectively). Likewise, neither peer pressure (“because I feel I am influenced by peer pressure”; 4.5–14.9%), nor pressure from coaches (“because of pressure from coaches/trainers”; 0–4.5%) were commonly reported reasons for consuming caffeinated products. Caffeinated products were not commonly reported as being used “to replace food or meals” (0–25.8%; caffeine tablets and chocolate respectively). 

### 3.4. Motivations for Not Consuming Caffeinated Products

**Figure 1:**
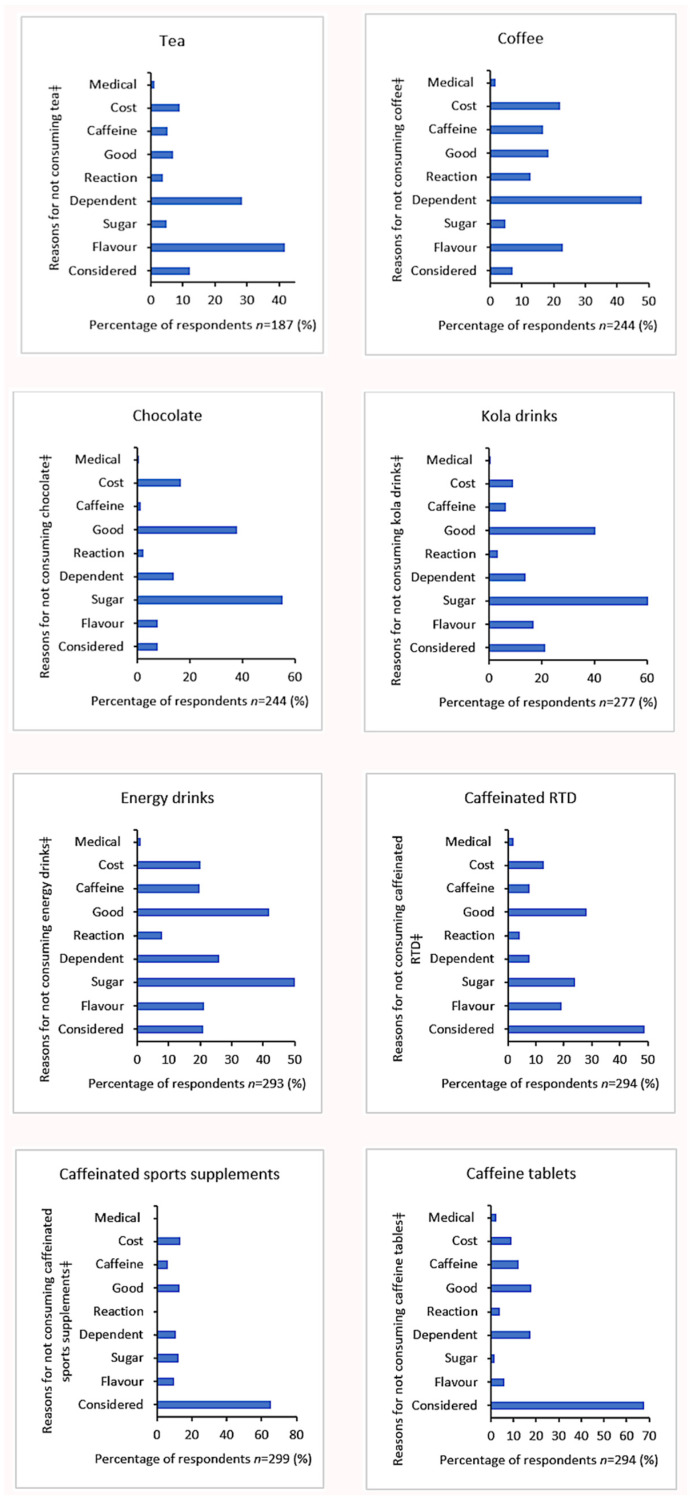
Reasons for not consuming caffeine-containing products; RTD: Ready-to-drink alcoholic beverage. Considered: “I have never considered taking it”; Flavour: “I don’t like the flavour”; Sugar: “There is too much sugar in it”; Dependent: “I don’t want to be dependent on it”; Reaction: “I react badly to it”; Good: “It isn’t ‘good’ for me”; Caffeine: “It has too much caffeine in it”; Cost: “It’s too expensive”; Medical: “I don’t consume it due to medical reasons.

Figure 1 highlights the reasons survey respondents gave for not consuming caffeine-containing products. The most common reason for the non-consumption of caffeinated products related to potential health concerns. The high sugar content (“It has too much sugar in it”) was a key concern relating to chocolate (55.3%), cola drinks (60.3%), energy drinks (50.2%) and caffeinated RTDs (24.1%). Others reported that they did not consume coffee (48.1%) and tea (28.3%) because “I don’t want to be dependent on it”. Some respondents chose not to consume certain products because they considered “it isn’t ‘good’ for me” (energy drinks, 42%; cola drinks, 40.4%; chocolate, 38.1%; and caffeinated RTDs, 28.2%). 

Hedonic aspects also affected consumption choice, with many tertiary students choosing not to consume certain caffeinated products because they did not like the flavour (tea, 41.7%; coffee, 23%; and caffeinated RTDs, 19.4%).

## 4. Discussion

The present study examined the motivations for caffeine consumption of a convenience sample of more than 300 NZ tertiary students. The results indicated that the key motivations for consumption of caffeinated products in this group of tertiary students varied according to product, but it included hedonic (sensory aspects e.g., taste and temperature), functional (e.g., increasing wakefulness, energy, and physical performance), and sociocultural factors (e.g., social situations, environment). These results were similar to the findings in a study of college students in Korea [30]. However, it is possible that tertiary students’ motivations for consuming specific products will vary from country to country due to cultural differences.

Research overseas suggests co-ingestion of caffeine and alcohol is widespread and increasing, particularly in U.S. tertiary student populations, where up to 28% reported such co-ingestion at least once in the past month [12,31,32]. However, there is currently limited research available on the prevalence of co-ingesting alcohol and caffeine in NZ tertiary students. In this convenience sample of NZ tertiary students, 38.5 % (*n* = 122) of the participants reported co-ingesting caffeine and alcohol; the majority co-ingested cola drinks and alcohol (71.3%), and approximately half consumed caffeinated RTDs (47.5%) and/or co-ingested energy drinks and alcohol (48.4%). Our previous study [23] showed that caffeinated RTD consumption was associated with an increased likelihood of exceeding the adverse-effect level (1.91 times) and the suggested safe limit (2.26 times) for caffeine. This information reinforces the validity of international concern regarding the concomitant consumption of caffeine and alcohol, which has resulted in multiple countries, including NZ, releasing warning statements about the health risks of co-ingesting alcohol and energy drinks [32,33]. Furthermore, Arria [34], reported that weekly or daily energy- drink consumption was strongly associated with alcohol dependence and thus reinforced the need for caffeine-related risk-reduction strategies. Knowledge of the motivations for co-ingesting caffeine and alcohol may be useful for developing such risk-reduction strategies.

It is important to understand the motivations for caffeine consumption and the misconceptions regarding its use. A previous study indicated tertiary students believed that caffeine could be used as a substitute for sleep, that caffeine increased short-term memory and alcohol tolerance, and that caffeine withdrawal could result in aggression and forgetfulness [35]. Since we observed that functional qualities (e.g., staying awake and waking up) were strong motivators for coffee, energy-drink, and caffeine-tablet consumption, it is important to improve consumers knowledge surrounding caffeine’s actual effects on both sleep and memory.

In NZ, any food containing caffeine from a natural source (except guarana) is exempt from caffeine-labelling requirements. This, paired with the variation in caffeine content of products that naturally contain caffeine, is a significant issue, as consumers have little-to-no information on which to base their decisions regarding the consumption of caffeine-containing products, potentially placing some individuals at increased risk of caffeine’s adverse effects [36]. The development and evaluation of educational interventions to enhance “caffeine literacy’’ in NZ is much needed. Within this context, we defined caffeine literacy as the knowledge of the caffeine content of different products, and the potential for variability in similar products, as well as the understanding of the variability of the individual positive and negative responses to consumption of different amounts of caffeine. Variability in similar products, refers to the fact that the amounts of caffeine in for example, a cappuccino, can vary, not only from outlet to outlet, but also within the same outlet, when made at different times and/or on different days.

Our previous study [23] reported that ingestion of caffeine tablets, coffee, caffeinated RTDs, and tea increased the likelihood of exceeding the adverse-effect level for caffeine (3 mg·kgbw^−1^·day^−1^), with consumption of coffee and caffeinated RTDs also associated with the likelihood of exceeding the suggested safe limit of 400 mg·day^−1^. The key motivators for both caffeine-tablet consumption and coffee were related to the functional qualities (energy, wakefulness), with the hedonic and sociocultural aspects also featuring for coffee. The consumption of RTDs was motivated by the sociocultural aspects as well as the functional (e.g., alcohol content), while tea was consumed for both hedonic and sociocultural reasons. To better educate caffeine consumers and lower their risk of over consumption of caffeine, distinct strategies may be required according to whether consumption is driven primarily by functional qualities versus hedonic and sociocultural reasons. Education to improve knowledge around caffeine’s true beneficial effects and to dispel misconceptions may be of greatest benefit to those who consume caffeine primarily for its functional effects while those who enjoy caffeinated beverages for their hedonic and social aspects may benefit most from more general information related to the caffeine content of products, variability of content in products, and potential side effects from overconsumption. In either case, educational resources and additional labelling to improve knowledge around caffeine consumption are required. 

The current study provides reasons for the consumption or non-consumption of caffeine-containing products individually rather than collectively. This is important, because not only do different products contain different levels of caffeine but they can also contain different ingredients that may also impact consumers’ reasons for or against consumption (e.g., sensory and social aspects). 

This study was subjected to limitations due to its cross-sectional design, the convenience sampling with voluntary participation, the potential for recall bias, and the small sample size particularly in the older age group. Moreover, as the motivation data were pooled any differences in perception due to gender were not investigated. Among the tertiary population, caffeine-consumption habits and motivations for consumption may change during the year in relation to periods of higher academic stress (e.g., exam period). One study showed 63% of tertiary students tended to increase their caffeine consumption during exam periods [37]. Hence, the results reported here did not necessarily reflect caffeine consumption throughout the entire academic year. Another limitation of the present study was that some ethnic groups were underrepresented (Māori—5.4% study sample vs. 22.4% tertiary student population; Pacific Peoples—6.9% study sample vs. 9.9% tertiary student population), whilst others are overrepresented (Asian—40.4% study sample vs. 15.5% tertiary student population [38]. Ethnicity has been known to influence food and beverage choices [39,40], and therefore, the results may not be representative of the true population, nor may they be applicable to other countries due to cultural differences. Another possible limitation was that participants may not have realised that advertising had affected their choice to consume a caffeinated beverage, because the effect had been too subtle. 

Research suggests that in order to reduce the risk of substance-related harm (such as caffeine intoxication), it is important to have an understanding of the consumers’ motivations for its use. The present study provides useful information for multiple stakeholders (e.g., the scientific community, public health professionals, regulatory agencies, consumers, retailers and the food industry) regarding to the motivations behind caffeine consumption by tertiary students in NZ. Armed with this information, strategies can be implemented (e.g., improved labelling, consumer education, additional regulations etc.) to increase caffeine literacy, which may help to ameliorate caffeine-related risk in this population group. 

## 5. Conclusions

In summary, 99% of this convenience sample of more than 300 NZ tertiary students regularly consumed caffeine, with the primary reason being for functional aspects of increased wakefulness and enhanced energy. The information from this study contributes to understanding the motivations behind caffeine consumption in NZ tertiary students and provides knowledge on which to develop strategies to reduce risk of excess caffeine consumption in this group. Motivations for consumption of the different caffeine-containing products vary and include functional qualities (e.g., coffee, RTDs), and sociality (chocolate, tea) and hedonic (tea) factors, as do those for non-consumption. Interestingly, participants did not identify advertising as a significant factor that influenced their consumption of caffeinated products. Consumer education to improve caffeine literacy, including caffeine’s true effects (benefits and side effects) as well as more general information on caffeine content and variability, along with product labelling, should be considered to reduce caffeine-related risks in NZ tertiary students. 

## Figures and Tables

**Table 1:** Participant socio-demographic characteristics.

Variable	Participants, *n* = 317; *n* (%)
Sex	
Male	148 (46.7)
Female	169 (53.3)
Age group	
16–18 years	51 (16)
19–30 years	236 (74.4)
31–50 years	25 (7.9)
51+ years	5 (1.6)
Ethnicity ^1^	
NZ European	150 (47.3)
Other European	56 (17.7)
Māori	17 (5.4)
Asian	128 (40.4)
Pacific Peoples	22 (6.9)
Middle Eastern/Latin American/African	16 (5.0)
Living situation	
Living alone	23 (7.4)
Living with family	174 (54.9)
Flatting with others	108 (34.1)
Halls of residence	7 (2.2)
Living with partner	5 (1.6)
Employment status	
No paid employment	211 (66.6)
Part-time employment	102 (32.2)
Full-time employment	4 (1.3)

^1^ Percentage exceeds 100 as participants were able to select more than one ethnicity. Adapted from [23], with permission from Stachyshyn, S.; Ali, A.; Wham, C.; Knightbridge-Eager, T.; Rutherfurd-Markwick, K., 2021.

**Table 2:** Comparison of consumption of caffeine sources by gender and age group.

Caffeine Source	Total, *n* = 317; *n* (%)	Male, *n* = 148; *n* (%)	Female, *n* = 169; *n* (%)	Pearson Chi-Square Value (χ^2^) ^a^	*p*-Value ^a^	16–18 Years, % (*n* = 51)	19–30 Years, % (*n* = 236)	31–50 Years, % (*n* = 25)	51+ Years, % (*n* = 5)	Pearson Chi-Square Value (χ^2^) ^b,e^	*p*-Value ^b,e^
Tea	227 (71.6)	92 (62.2)	135 (79.9)	12.185	<0.001	66.7	71.6	80	80	-	0.699
Coffee	242 (76.3)	113 (76.3)	137 (81.1)	4.473	0.034	68.6	77.1	84	80	-	0.440
Chocolate	259 (81.7)	111 (75)	148 (87.6)	8.345	0.004	86.3	80.5	84	80	-	0.788
Cola drinks	156 (49.2)	78 (52.7)	78 (46.2)	1.354	0.245	45.1	51.3	44	20	-	0.469
Energy drinks	128 (40.4)	67 (45.3)	61 (36.1)	2.759	0.097	35.3	44.1	20	20	-	0.066
Caffeinated RTD	58 (18.3)	29 (19.6)	29 (17.2)	0.313	0.576	21.6	19.1	8	0	-	0.409
CCS supplement	21 (6.6)	14 (9.5)	7 (4.1)	2.798 ^c^	0.094 ^c^	0	8.5	4	0	-	0.118
Caffeine tablets	10 (3.2)	4 (2.7)	7 (4.1)	0.153 ^c^	0.696 ^c^	0	4.7	0	0	-	0.356
None	3 (0.9)	2 (1.4)	1 (0.6)	-	0.6 ^d^	2	0.8	0	0	-	0.589

RTD: ready-to-drink alcoholic beverage; CCS supplement: caffeine-containing sports supplement; - Fisher’s exact test, no value available; ^a^ Values based on male vs female; ^b^ Values based upon age groups; ^c^ Yates continuity correction (minimum expected count < 10); ^d^ Fisher’s exact test (minimum expected count < 5); ^e^ Fisher’s exact test (more than 20% of expected counts < 5).

**Table 3:** Co-ingestion of alcohol with caffeine and energy drinks by participant demographic and characteristics.

Participant Demographic/Characteristic	Participants Who Co-Ingest Caffeine and Alcohol (%)	Pearson’s Chi-Square Value (χ^2^)	*p*-Value	Participants Who Co-Ingest Alcohol and Energy Drinks (%)	Pearson’s Chi-Square Value (χ^2^)	*p*-Value
Gender						
Male (*n* = 148)	38.5	0	0.990	21.6	1.66	0.198
Female (*n* = 169)	38.5			16		
Age group						
16–18 years old (*n* = 51)	41.2	7.144 ^a^	0.059 ^a^	21.6	4.862 ^a^	0.169 ^a^
19–30 years old (*n* = 236)	40.7			19.9		
31–50 years old (*n* = 25)	20			4		
51+ years old (*n* = 5)	0			0		
Living situation						
Living alone (*n* = 23)	30.4	4.202 ^a^	0.379 ^a^	13	1.430 ^a^	0.857
Living with family (*n* = 174)	36.8			18.4		
Flatting with others (*n* = 108)	40.7			21.3		
Hall of residence (*n* = 7)	71.4			14.3		
Living with partner (*n* = 5)	40			0		
Working status						
Paid work (*n* = 211)	47.2	5.07	0.020	30.2	14.09	< 0.001
No paid work (*n* = 106)	34.1			12.8		
Smoking status						
Smokes (*n* = 47)	63.8	15.1	<0.001	40.4	17.082	<0.001
Does not smoke (*n* = 270)	34			14.9		

^a^ Fisher’s exact test (Minimum expected count < 5).

**Table 4:** Most common reasons *^,^^#^ for consumption of caffeine-containing products.

Reasons for the Consumption	Accumulative % of Agreement on a Four-Point Likert Scale
Tea (*n* = 227)	
“for the warmth”	92.6%
“for the taste”	89.5%
“to comfort and relax myself”	86.9%
“because it is easily available”	80.8%
“whenever one is offered to me”	79.9%
“with family”	73.8%
“with friends”	61.6%
Coffee (*n* = 242)	
“to stay awake”	86.8%
“for the warmth”	86.3%
“to wake up”	85.9%
“for mental energy”	85.5%
“for the taste”	85.1%
“for energy”	84.3%
“with friends”	83.0%
Chocolate (*n* = 259)	
“for the taste”	95.4%
“as a treat or luxury food”	88.8%
“to comfort and relax myself “	79.6%
“with friends”	77.6%
“whenever it is offered”	77.2%
“with family”	72.9%
“for the warmth (drinking chocolate)”	71.0%
Cola drink (*n* = 156)	
“because they are cold and refreshing”	90.6%
“for the taste”	89.3%
“with takeaway food”	85.5%
“with friends”	78.0%
“as a treat drink”	75.4%
“because it is easily available”	67.3%
“while travelling”	67.3%
Energy drink (*n* = 128)	
“for energy”	90.6%
“to stay awake”	89.1%
“to wake up”	85.2%
“for mental energy”	84.3%
“for physical energy”	70.3%
“because they are cold and refreshing”	66.5%
“for the taste”	65.7%
Caffeinated ready-to-drink alcoholic beverage (*n* = 58)	
“with friends”	91.8%
“for the alcohol content”	85.2%
“because others are drinking them”	78.7%
“whenever one is offered to me”	77.1%
“because I know how much alcohol is in them”	72.1%
“for the taste”	70.5%
“because they are cheaper than other alcoholic drinks”	62.3%
Caffeine-containing sports supplements (*n* = 21)	
“to improve physical performance”	86.3%
“for energy”	86.3%
“for physical energy”	81.8%
“as they are convenient to take”	59.1%
Caffeine tablets (*n* = 10)	
“for energy”	90.9%
“for mental energy”	90.9%
“to stay awake”	81.8%
“to wake up”	81.8%
“as they are convenient to take”	63.7%
“for physical energy”	54.6%

* The seven most common choices are shown, except for those with low percentage agreement; then only the choices that exceeded 50% are shown; ^#^ Other choices included “because it’s cheaper than other hot drinks”, “because it’s what I drink with food”, “out of boredom”, “because I feel I am influenced by peer pressure”, “out of habit”, “when I am stressed”, “because I feel I am influenced by advertising”, “as my culture influences me to drink it”, and “to replace food or meals”. For tea: “when I have had enough coffee for the day” and “because I think coffee has too much caffeine in it”. For coffee and energy drinks: “when I am smoking”. For coffee, energy drinks, and caffeine tablets: “while driving long distances”. For chocolate: “because it is already in many of the foods I eat” and “more when I am on my period” (people who menstruate). For cola drinks: for the bubbles/ how it feels in my mouth” and “instead of coffee when the weather is hot”. For cola drinks and energy drinks: “instead of alcohol” and “as a mixer for alcohol”. For caffeinated RTD: “because they are easy to transport” and “instead of spirits”. For caffeine-containing sports supplements and caffeine tablets: “because of pressure from coaches/trainers”, “because they are easy to transport”, and “as a substitute for illegal drugs”.

## Data Availability

The data presented in this study are available on request from the corresponding author.
